# High magnitude of under nutrition among HIV infected adults who have not started ART in Tanzania--a call to include nutrition care and treatment in the test and treat model

**DOI:** 10.1186/s40795-017-0180-0

**Published:** 2017-08-03

**Authors:** Bruno F. Sunguya, Nzovu K. Ulenga, Hellen Siril, Sarah Puryear, Eric Aris, Expeditho Mtisi, Edith Tarimo, David P. Urassa, Wafaie Fawzi, Ferdnand Mugusi

**Affiliations:** 1 Afya Bora Consortium, School of Public Health and Social Sciences, Muhimbili University of Health and Allied Sciences, 9, United Nations Road, Upanga West, Dar es salaam, Tanzania; 2 Management and Development for Health, Dar es salaam, Tanzania; 3 African Academy for Public Health, Dar es salaam, Tanzania; 4 Department of Global Health, University of Washington, Seattle, WA USA; 5 Departments of Global Health and Population, Nutrition, and Epidemiology, Harvard T.H. Chan School of Public Health, Boston, MA USA

**Keywords:** Undernutrition, Antiretroviral therapy, Nutrition care, And integrated care

## Abstract

**Background:**

Undernutrition among people living with HIV (PLWHIV) can be ameliorated if nutrition specific and sensitive interventions are integrated into their HIV care and treatment centers (CTC). Integrated care is lacking despite expansion of antiretroviral therapy (ART) coverage, representing a substantial missed opportunity. This research aims to examine nutritional status and associated risk factors among HIV-positive adults prior to ART initiation in Tanzania in order to characterize existing gaps and inform early integration of nutrition care into CTC.

**Methods:**

We analyzed data from 3993 pre-ART adults living with HIV enrolled in CTCs within the Trial of Vitamin (TOV3) and progression of HIV/AIDS study in Dar es salaam, Tanzania. The primary outcome for this analysis was undernutrition, measured as body mass index (BMI) below 18.5 kg/m^2^. We conducted descriptive analyses of baseline characteristics and utilized multiple logistic regression to determine independent factors associated with pre-ART undernutrition.

**Results:**

Undernutrition was prevalent in about 27.7% of pre-ART adults, with a significantly higher magnitude among males compared to females (30% vs. 26.6%, *p* < 0.025). Severe undernutrition (BMI < 16.0 kg/m^2^) was prevalent in one in four persons, with a trend toward higher magnitudes among females (26.2% vs. 21.1% *p* = 0.123). Undernutrition was also more prevalent among younger adults (*p* < 0.001), those with lower wealth quintiles (*p* = 0.003), and those with advanced HIV clinical stage (*p* < 0.001). Pre-ART adults presented with poor feeding practices, hallmarked by low dietary diversity scores and infrequent consumption of proteins, vegetables, and fruits. After adjusting for confounders and important co-variates, pre-ART undernutrition was associated with younger age, low wealth indices, advanced clinical stage, and low dietary diversity.

**Conclusions:**

One in every four pre-ART PLWHIV presented with undernutrition in Dar es salaam, Tanzania. Risk factors for undernourishment included younger age, lower household income, advanced HIV clinical stage, and lower dietary diversity score. Knowledge of the prevalence and prevailing risk factors for undernutrition among pre-ART PLWHIV should guide targeted, early integration of nutrition interventions into routine HIV care and treatment in high-prevalence, low-income settings such as Tanzania.

## Background

Massive gains have been achieved in the fight against HIV/AIDS owing to the global efforts in rolling out effective interventions [1]. These include treatment with potent antiretroviral drugs, strategies to prevent mother to child transmission, advocacy, and other behavior modification interventions. Such interventions have led to a significant decline in HIV prevalence in the most affected countries in sub Saharan Africa—Tanzania is no exception [2]. For example, the prevalence of HIV among adults aged 15–49 in Tanzania has declined from 7.0% in 2004 [3] to 5.3% in 2012 [4], and 4.7% in 2015. Moreover, the incidence of the disease has also declined to below 1% of the general population [5].

The decline in HIV prevalence in Tanzania is partially attributable to increased ART coverage. The cumulative number of people on ART in Tanzania increased by 50% in only 1 year between 2011 to 2012 [4]. By 2015, about 47.6% of people living with HIV (PLWHIV) were on ART [5]. However, even with such efforts, HIV case fatality rate has remained high. A total of 47,860 people died of disease in 2015 alone [5]. Such poor treatment outcomes have been attributed to poor viral suppression, continued immune suppression, poor nutritional status, opportunistic infections, and non-communicable diseases owing to chronic HIV/AIDS [6]. Tailor-made interventions are therefore needed to address modifiable factors and should be integrated into their routine HIV care and treatment.

Improving nutritional status may be an entry point in addressing many other risk factors among PLWHIV. Evidence has shown that food insecurity results in poor outcomes among PLWHIV [7]. The most commonly demonstrated negative outcomes have been nutritional status, adherence to medication, and psychological wellbeing [8]. In Tanzania, poor feeding practices are widespread and have been associated with undernutrition [9, 10]. Despite this evidence, nutrition integrated HIV care is lacking in Tanzania, representing a substantial missed opportunity. Beginning in 2016, the country adopted the test and treat strategy for HIV, which will result in more people on ART and longer durations of treatment. For this strategy to attain its desired effects, HIV-positive adults should also be provided with nutrition care and treatment that is integrated into their routine HIV care and treatment.

To design tailor made interventions for adults living with HIV, it is essential to understand the magnitudes and factors associated with undernutrition in Tanzania. Such evidence has not been published. The aim of the current study was therefore, first, to examine magnitudes and severity of undernutrition among adults enrolled for ART services. The second aim was to examine determinants of undernutrition in such population. Results from this study aimed to inform the HIV/AIDS program on the needs to integrate other important but left out services to improve treatment outcomes among adults attending care and treatment services in Tanzania and countries with similar HIV/AIDS landscape.

## Methods

### Study design

This cross-sectional study analyzed secondary data of the baseline phase of a randomized double-blind controlled trial on the efficacy of multivitamins on disease progression among HIV-positive adults in Dar es salaam, Tanzania—the TOV3 trial. We used this data to study ART-naïve adults so as to inform of the needs for integrated nutrition care on ART services. The trial aimed at recruiting a total of 4000 randomized into two intervention arms. Data were collected before ART initiation and the participants were followed for 5 years.

### Study settings

Participants were recruited from seven HIV care and treatment clinics (CTCs). At the time of study preparation, Highly Active Antiretroviral Therapy (HAART) was provided under the Tanzania National AIDS Control Program with the support of the President’s Emergency Plan for AIDS Relief program and in collaboration with the Harvard School of Public Health, Muhimbili University of Health and Allied Sciences, and the City of Dar es Salaam Regional Office of Health in only seven CTCs. Those CTCs were Infectious Disease Center (IDC), Buguruni, Mwananyamala, Temeke, Mbagala-Rangitatu, Amana, and Sinza CTCs. A CTC is the primary health facility for care and treatment of PLWHIV. Therefore, all CTCs were included as study sites for the TOV3 study. HIV-positive individuals are referred to the CTCs after testing positive in the community, voluntary care and treatment facilities, or any other health facility.

### Population and sampling

The TOV3 study included all confirmed HIV-positive patients who had not started ART and consented to participate. Participant eligibility was determined at the first CTC visit. HIV status was confirmed using two licensed HIV-1 Enzyme-linked immunosorbent assay (ELISA) or two rapid tests, and discordant results were verified via Western Blot. Exclusion criteria included individuals aged below 18 years, and those who would be on transit. For the current paper, we selected adult participants with no missing outcome variables of interest.

### Measurements

The outcome variable of interest in the current study was undernutrition measured through BMI in kg/m^2^. BMIs were calculated based on height and weight at the first study visit. Undernutrition was defined as BMI of less than 18.5 kg/m^2^. Severity of undernutrition was further classified into severe (BMI < 16 kg/m^2^), moderate (BMI 16 to <17 kg/m^2^) and mild (BMI 17 to <18.5 kg/m^2^).

Independent variables included socio-demographic characteristics, feeding practices variables, psychosocial variables, and HIV clinical stage. For demographic characteristics, variables included age (in years), sex, the highest attained level of education, and socio economic status. Dietary characteristics included dietary diversity score. Psychosocial variables included emotional distress score and depression score. We used the World Health Organization’s HIV clinical classification to assess the disease progression at the time of recruitment.

Education level was categorized into primary school incomplete, primary school complete, secondary school attended, and advanced education attended. Age in years was divided into quintiles to distribute the age groups evenly. Economic status was assessed using weighted wealth index. It incorporated household assets ownership, housing characteristics, fuel for lighting and cooking, type of toilet, source of water, and feeding characteristics. Dichotomous variables were constructed and factor analysis using principle component analysis (PCA) was used to reduce 31 items to 14 in component one to three. These were the most important components before the hinge of the scree plot. Factor loadings were used as item weights totaled to yield the wealth index for each household. It was then divided into quintiles designating lowest (−1.56–1.40), low (1.41–2.19), middle (2.20–2.71), high (2.72–3.50) and highest (3.51–4.85) quintiles of the economic status.

Dietary diversity scores were calculated by summing the number of food groups consumed in the day that preceded the enrolment. It was used as a continuous variable and a categorical variable. For the categorical variable, dietary diversity score below three was considered low. Food groups assigned were starch or carbohydrate, any protein, animal protein, plant protein, green vegetables, and fruits. Categorical variables were made for each food groups as none, once, 2–3, and 4 and above times a day.

We measured emotional distress using the Hopkins Symptom Checklist (HSCL), which assigns scores for depression (15 items) and anxiety (10 items). In the current study, the 15-item depression scale had a Cronbach’s alpha of 0.9168. The Cronbach’s alpha for the 10-item anxiety scale was 0.8871. The 25-item HCL checklist had a Cronbach’s alpha of 0.9333. Finally, we used the WHO clinical HIV classification to stage the disease progress among the enrollees. Stage one and two are the early stages whereas three and four signify advanced disease.

### Data analysis

Descriptive and regression analyses were used. Chi-square test was used to compare the characteristics of participants by gender and nutritional status for categorical variables. T-test was used for continuous variables. We conducted bivariate logistic regression to examine the associations between each independent variable and undernutrition. Statistical associations with *p*-values below or equal to 0.2 were entered into a multiple logistic regression model to find factors associated with undernutrition after adjusting for confounders and important covariates. Social support and emotional distress were not entered into the model because of their high correlations with depression score. Statistical significance was set at the *p-value* < 0.05. Missing variables were excluded as cases during regression analyses when the variables introduced into the modules were being analyzed. Analyses were conducted using Stata version 13.

### Ethical consideration

The current study utilized data from the Trial of Vitamin study (TOV3) that observed all ethical guidelines. The parent study was approved by the Institutional Review Board of the National Medical Research Institute (NIMR), Muhimbili University of Health and Allied Sciences (MUHAS), and the Harvard School of Public Health. Participants were assured of confidentiality and anonymity throughout the process and for all reports and publications generated. A written consent was obtained after the participants were explained about the study and agree to voluntarily participate. Participation was voluntary and there were no implications for care at the CTC upon refusal to participate.

## Results

Data of 3993 HIV-positive adults were available for analyses. Of them, 1268 (31.8) were male. The mean age at enrolment to the TOV3 study was 38.0 years, sd. 8.6.

### General characteristics

Male participants had a higher mean age in years compared to their female counterparts (40.9 vs. 36.7, *p* < 0.001). Table 1 shows participants’ characteristics. A higher proportion of male participants had higher weighted wealth index compared to female. For example, only 15.2% of males were in the lowest wealth quintile compared to 22.1% of female participants (*P* < 0.001). Highest attained level of education was also different across the gender profiles. There was no difference in the proportion of participants with the primary level of education (*p* = 0.949). However, a higher proportion of male participants had attended any secondary school class compared to females (51.8% vs. 40.2%, *p* = 0.001) and a similar trend was found for advanced schooling (29.6% vs. 17.7%, *p* < 0.001).

**Table 1:** General characteristics of TOV study participants

Variable	Male		Female		Total		*p*
	N (x̅)	% (SD)	N (x̅)	%(SD)	N	%	
*Socio-demographic characteristics*							
Wealth index							
Lowest	151	15.2	498	22.1	649	20.0	<0.001
Low	177	17.82	480	21.3	657	20.2	
Middle	242	24.4	492	21.8	734	22.6	
High	215	21.7	413	18.3	628	19.4	
Highest	208	20.9	370	16.5	578	17.8	
Primary school							
Completed	743	86.5	1659	86.4	2402	86.4	0.949
Not completed	116	13.5	261	13.6	377	13.6	
Secondary education							
Any class	170	51.8	254	40.2	424	44.2	0.001
None	158	48.2	378	59.8	536	55.8	
Advanced schooling							
Any level	73	29.6	89	17.7	162	21.6	<0.001
None	174	70.4	415	82.3	589	78.4	
*Nutritional status (BMI)*							
Nutritional status							
Underweight	380	30.0	724	26.6	1104	27.7	<0.001
Normal	769	60.7	1506	55.3	2275	57.0	
Overweight	119	9.3	495	18.1	614	15.3	
Severity of undernutrition							
Severe	80	21.1	190	26.2	270	24.4	0.123
Moderate	81	21.3	157	21.7	238	21.6	
Mild	219	57.6	377	52.1	596	54.0	
Feeding practices							
Dietary diversity							
Mean	(2.94)	(0.05)	(2.94)	(0.03)			0.424
DDS < 3	459	46.2	985	46.1	1444	46.1	0.945
DDS > =3	534	53.8	1152	53.9	1686	53.9	
Any starchy							
None	93	9.5	182	8.5	275	8.8	
Once	833	83.9	1782	83.4	2615	83.6	0.294
2–3	56	5.6	155	7.2	211	6.7	
4 and above	11	1.0	18	0.9	29	0.9	
Any protein							
None	299	30.1	677	31.7	976	31.2	
Once	647	65.2	1355	63.4	2002	64.0	0.367
2–3	35	3.5	89	4.1	124	3.9	
4 and above	12	1.2	16	0.8	28	0.9	
Any Animal protein							
None	313	31.5	711	33.3	1024	32.7	
Once	634	63.9	1332	62.3	1966	62.8	0.180
2–3	34	3.4	82	3.8	116	3.7	
4 and above	12	1.2	12	0.6	24	0.8	
Any other protein							
None	870	87.6	1884	88.2	2754	88.0	
Once	119	12.0	228	10.7	347	11.1	0.144
2–3	3	0.3	20	0.9	23	0.7	
4 and above	1	0.1	5	0.2	6	0.2	
Any green vegetables							
None	605	60.9	1265	59.2	1870	59.8	
Once	350	35.3	778	36.4	1128	36.0	0.673
2–3	31	3.1	81	3.8	112	3.6	
4 and above	7	0.7	13	0.6	20	0.6	
Any fruits							
None	456	47.9	999	46.8	1455	46.5	
Once	470	47.3	979	45.8	1449	46.3	0.348
2–3	51	5.2	135	6.3	186	5.9	
4 and above	16	1.6	24	1.1	40	1.3	

A total of 1104 (27.7%) had a BMI score below 18.5 kg/m^2^. A higher proportion of them were male compared to female participants (30.0% vs. 26.6%, *p* < 0.001. Of those with undernutrition, 24.4% were severe while 21.6% were moderate and 54.0% were mild.

The mean dietary diversity score was 2.94 for both sexes. About 46.1% of all patients consumed less than 3 different types of foods in the day that preceded the interview. As high as 91.2% of recruited clients consumed starchy foods at least once in the previous day, while only 68% consumed any type of food with protein at least once during the same period. Animal sources of protein were consumed by about 67% of those interviewed; only 12% reported consuming plant related protein-contained foods. About 60% did not consume any green vegetables and 46.5% did not consume any type of fruit.

### Descriptive characteristics according to nutritional status

Table 2 displays the results of descriptive analyses stratified by undernutrition. Younger age was associated with undernutrition. Mean age among individuals with undernutrition was lower compared to those with normal or higher BMI (37.14 sd. 8.59 vs. 38.61 sd. 8.61, *p* < 0.001). The trend of undernutrition was such that, young ages had higher magnitudes of undernutrition compared to higher age groups, *p* = 0.003. Women had a lower magnitude of undernutrition compared to their male counterparts (30.0% vs. 26.6%, *p* = 0.025). As expected, wealth index was associated with undernutrition. The lower weighted wealth index quintiles had higher magnitudes of undernutrition, while as the weighted wealth index increased, the BMI tended to increase, *p* < 0.001.

**Table 2:** Descriptive analyses, BMI as outcome variable

Variable	BMI < 18.5		BMI > =18.5		Total		*p*
	N (x̅)	% (SD)	N (x̅)	%(SD)	N	%	
Age (mean years)	(37.14)	(8.59)	(38.32)	(8.61)	3999		<0.001
Age (years)							
18.47–30.65	249	22.6	551	19.0	800	20.0	0.003
30.66–34.96	234	21.2	566	19.6	800	20.0	
34.97–39.07	231	20.9	569	19.7	800	20.0	
39.08–44.80	204	18.5	596	20.6	800	20.0	
44.80–83.35	186	16.8	613	20.1	799	20.0	
Sex							
Male	380	30.0	888	70.0	1268	31.8	0.025
Female	724	26.6	2001	73.4	2725	68.2	
Wealth index							
Lowest	217	26.6	434	17.8	651	20.0	<0.001
Low	196	24.0	462	19.0	658	20.2	
Middle	175	21.5	560	23.0	735	22.6	
High	123	15.1	506	20.8	629	19.4	
Highest	105	12.8	473	19.4	578	17.8	
Emotional distress	(1.29)	(0.43)	(1.25)	(0.39)	3055		0.019
Depression score	(1.35)	(0.52)	(1.30)	(0.48)			0.010
Depression							
Yes	779	70.4	1849	63.6	2628	65.5	<0.001
No	327	29.6	1057	36.4	1384	34.5	
Social support	(25.37)	(8.00)	(25.07)	(8.39)	3154		0.381
Alcohol dependency							
Yes	4	1.4	17	2.1	21	1.9	0.005
No	290	98.6	797	97.9	1091	98.1	
HIV clinical stage							
Stage 1	12	1.2	181	6.8	193	5.3	<0.001
Stage 2	112	11.1	522	19.5	634	17.2	
Stage 3	599	59.4	1701	63.8	2300	62.6	
Stage 4	285	28.3	264	9.9	549	14.9	
Dietary diversity							
Mean	(2.90)	(1.56)	(2.97)	(1.61)	3137		0.280
DDS < 3	399	48.7	1050	45.3	1449	46.2	0.099
DDS > =3	421	51.3	1267	54.7	1688	53.8	

There were significant differences in psychosocial variables among those with low compared to those with normal or higher BMI. For example, mean emotional distress was higher among individuals with undernutrition compared with their counterparts (1.29 sd. 0.43 vs. 1.25 sd. 0.39, *p* = 0.019). Mean score in the depression scale was also higher among individuals with undernutrition compared to their counterparts (1.35 sd. 0.52 vs. 1.30 sd. 0.48, *p* = 0.010). Magnitude of depression was high in this population. For example, about 65.5% of the enrolled HIV-positive individuals had depression symptoms. A higher proportion was among individuals with undernutrition compared to their counterparts (70.4% vs 63.6%, *p* < 0.001). There was no difference in social support indicators between individuals with or without undernutrition (*p* = 0.381). A total of 4 (1.4%) individuals with alcohol dependency had undernutrition compared to 17(2.1%) who had normal nutritional status (*p* = 0.005).

HIV clinical stage was also associated with undernutrition. For example, only 1.2% of individuals with stage 1 were underweight status compared to 59.4% of those with stage 3 and 28.3% of those with stage 4 disease. There was no statistically significant difference in dietary diversity among those with undernutrition and their counterparts, however, a lower proportion of those with undernutrition (89.6%) had not consumed any of the starchy foods.

### Factors associated with undernutrition

Table 3 shows results of bivariate and multiple logistic regression analyses. At bivariate logistic regression analyses, association between age, sex, wealth index, depression, HIV clinical stage, and emotional distress with nutritional status reached a statistically significant level. Social support and alcohol dependency were not statistically significant in bivariate analyses. The multiple regression analysis model included all statistically significant variables, with the exception of emotional distress, which was excluded due to the high correlation with depression.

**Table 3:** Logistic regression analyses on the factors associated with low BMI among the clients enrolled into the study

Variable	Logistic regression			Multiple logistic regression		
	OR	95% CI	*P*-value	AOR	95% CI	*P*-value
Age (quintiles)						
18.47–30.65	1.00			1.00		
30.66–34.96	0.91	0.74–1.13	0.414	0.84	0.63–1.12	0.241
34.97–39.07	0.90	0.73–1.11	0.326	0.84	0.63–1.13	0.259
39.08–44.80	0.76	0.61–0.94	0.013	0.66	0.49–0.88	0.005
44.80–83.35	0.67	0.54–0.84	<0.001	0.63	0.46–0.85	0.002
Sex						
Male	1.00			1.00		
Female	0.85	0.73–0.98	0.025	0.87	0.70–1.07	0.179
Wealth index						
Lowest	1.00			1.00		
Low	0.85	0.67–1.07	0.168	0.80	0.60–1.06	0.114
Middle	0.63	0.49–0.79	<0.001	0.67	0.50–0.88	0.004
High	0.49	0.38–0.63	<0.001	0.49	0.37–0.67	<0.001
Highest	0.44	0.34–0.58	<0.001	0.46	0.34–0.63	<0.001
Depression	1.23	1.05–1.44	0.010	1.15	0.96–1.38	0.138
HIV clinical stage						
Stage 1	1.00			1.00		
Stage 2	3.24	1.74–6.01	<0.001	5.05	1.99–12.83	0.001
Stage 3	5.31	2.94–9.58	<0.001	8.77	3.55–21.65	<0.001
Stage 4	16.28	8.87–29.90	<0.001	22.90	9.08–57.75	<0.001
Dietary diversity						
DDS < 3	1.00			1.00		
DDS > =3	0.87	0.75–1.03	0.099	0.79	0.66–0.96	0.016
Alcohol dependency	0.63	0.21–1.90	0.414			
Emotional distress	1.26	1.04–1.53	0.019			
Social support	1.00	0.99–1.01	0.300			

Compared to the first age quintile (below 30.6 years), those at the fourth quintile (39.1–44.8 years) were 34% less likely to have undernutrition (*p* = 0.005) after adjusted for other important confounders and covariates. Moreover, those at fifth age quintile (>44.8 years) were 37% less likely to have undernutrition compared to their counterparts in the first quintile of age (below 30.6 years), *p* = 0.002. Compared to HIV-positive males, females were 13% less likely to be underweight, but this association did not reach a statistically significant level (*p* = 0.179). Higher wealth index was also associated with better nutritional status. For example, after adjusting for confounders, individuals at middle wealth index were 33% less likely to have undernutrition compared with those at the lowest weighted wealth index (*p* = 0.004). Similarly, those at higher and highest wealth quintiles were 51% and 54% less likely to exhibit undernutrition compared with their counterparts at the lowest wealth index (*p* < 0.001).

The association between depression and undernutrition did not reach a statistical significant level. An increase in the HIV clinical stage was associated with higher magnitudes of undernutrition. For example, patients with HIV clinical stage two were five times more likely to have undernutrition compared to those with clinical stage 1 (*p* = 0.001). Those with HIV clinical stage 3 were 8.77 times more likely to be underweight compared to those with HIV clinical stage 1 (*p* < 0.001). Likewise, those at stage 4 were 22.90 times more likely to have undernutrition compared to those with stage 1 of the disease (*p* < 0.001). HIV-positive individuals who had a dietary diversity score of three or above were 21% less likely to be undernourished (*p* = 0.016).

## Discussion

This study examined magnitudes of undernutrition among pre-ART HIV-positive adults in Dar es salaam, Tanzania. The study found that, more than one in every four of pre-ART adults had undernutrition measured as BMI below 18.5 kg/m^2^. Of them, one in four had a severe form of undernutrition reflected by a BMI below 16.0 kg/m^2^. Those with undernutrition were more likely to be of the younger age, low wealth index, advanced HIV clinical stage, and low dietary diversity score.

More than one in four pre-ART HIV-positive adults in Dar es salaam presented with undernutrition. Such unprecedented magnitude of undernutrition is not uncommon in this [9, 11] and other areas with similar context [12]. A syndemic theory can help explain such high magnitude of undernutrition in this context. First, the disease itself can exacerbate nutritional debilitation through high-energy expenditure, loss through fever, recurrence of opportunistic infections, including diarrheas that lead to emaciation of lean tissues. Secondly, HIV as a disease can subject the affected people to socio-economic disadvantages including food insecurity, poverty, and other challenges associated with undernutrition [13]. People living with HIV are therefore at a higher risk of undernutrition compared to their counterparts in the general population.

Despite such burden, to a large extent, nutrition care and treatment has not been integrated into most HIV care and treatment facilities [14]. Tanzania is no exception. With such magnitudes of undernutrition, HIV-positive individuals are likely to succumb to treatment failure, higher risk of opportunistic infections [15, 16], and even mortality [11, 12, 17]. Addressing undernutrition among adults is of paramount importance to attain treatment goals, especially in the new era of test and treat for all HIV-positive individuals.

In this study, undernutrition was associated with a number of modifiable factors. Like in other studies conducted in similar settings among HIV-positive children [9, 10], adults were more likely to succumb to undernutrition when they had poor feeding practices such as consuming foods with low dietary diversity. Moreover, only a small proportion consumed protein-rich foods, fruits, or vegetables in their routine meals, similar to other studies [18–20]. Such low diversity diets predispose patients not only to macronutrient undernutrition but also to micronutrient deficits [8]. Poor feeding practices could also be associated with other social demographic disadvantages [21, 22]. Studies have revealed a link between poor feeding practices with food insecurity, poverty, and other social demographic characteristics [8]. However, in the context of Tanzania, even in areas with high yields of agricultural products, HIV-positive individuals succumb to selective food insecurity and therefore undernutrition [10]. Nutrition specific and sensitive interventions should be integrated within ART services to complement care for these populations [23]. Nutrition specific interventions include improving feeding practices such as frequency, diversity, and quantity. It also includes prevention and treatment of opportunistic infections leading to nutritional challenges such as diarrhea. Nutrition sensitive interventions include improving food insecurity, access to health care, poverty alleviation and education pertinent to food and nutrition.

Like in other contexts, undernutrition among pre-ART individuals was associated with low wealth index [10, 24]. Previous models have established associations between poverty and undernutrition, especially among PLWHIV [8]. For this specific population in Tanzania, such an association calls for establishment of a mechanism to identify those from poor households and to develop an innovative approach to alleviate the effects of poverty. In so doing, we can improve their undernutrition and, in turn, improve their HIV treatment outcomes [10]. Such poverty-alleviating interventions include conditional cash transfer [25, 26], small productivity groups, microfinancing of economic activities, and other livelihood programs [27, 28].

Advanced HIV clinical stage was also associated with undernutrition [12]. This should also be taken into consideration when patients are enrolled into care as they are more prone to mortality [12]. Early, integrated and innovative nutrition interventions can improve their outcomes and should be targeted at this vulnerable population.

To achieve better treatment outcomes, the results of this study emphasize the integration of nutrition care and treatment into HIV care and treatment [29, 30]. This should be done from the moment these individuals are enrolled into care and sustained throughout treatment to attain optimal results [31]. Individuals with depression were more likely to be undernourished in this study although not at the multivariate regression analyses. The high magnitude of depression also emphasizes the needs for integrated services to ameliorate HIV-positive individuals with psychological stress and depressive symptoms to further improve their quality of lives and treatment outcomes. Currently, HIV care and treatment centers do not possess such services in Tanzania.

Results of this study should be interpreted carefully owing to the following limitations. First, this was a secondary analysis of data originally collected for a trial of vitamins. It might therefore have failed to include all confounders that should have been controlled for, especially in regression analyses. Second, results of this study may not be generalizable to the entire country. However, they may be useful in areas with similar context as in Dar es Salaam. Third, we used atypical dietary diversity score. This could have introduced validity challenges of assessment of dietary diversity score of participants.

## Conclusion

The fact that more than one in four pre-HAART HIV-positive adults in Dar es salaam, Tanzania presented with undernutrition in various severity implies nutrition care is needed hand in hand with scaling up of the test and treat strategy. Such unprecedented magnitude of undernutrition was associated with young age, advanced clinical stage, low wealth index, and low dietary diversity. To ameliorate undernutrition and improve treatment outcomes with ART, integration of nutrition and treatment into HIV care and treatment is of paramount importance in Dar es salaam, and areas with similar context.

## Abbreviations

ABC: Afya Bora Consortium
AIDS: Acquired immunodeficiency syndrome
ART: Antiretroviral therapy
BMI: Body Mass Index
CTC: Care and treatment center
ELISA: Enzyme-linked immunosorbent assay
HAART: Highly active antiretroviral therapy
HIV: Human Immunodeficiency Virus
MUHAS: Muhimbili University of Health and Allied Sciences
NIH: National Institute of Health
NIMR: National Institute of Medical Research
PLWHIV: People living with Human Immuno-deficiency Virus
Sd.: Standard deviation
TOV3: Trial of Vitamin 3
